# Loading equine oocytes with cryoprotective agents captured with a finite element method model

**DOI:** 10.1038/s41598-021-99287-9

**Published:** 2021-10-06

**Authors:** Sercan Içli, Meisam Soleimani, Harriëtte Oldenhof, Harald Sieme, Peter Wriggers, Willem F. Wolkers

**Affiliations:** 1 Biostabilization Laboratory — Lower Saxony Centre for Biomedical Engineering, Implant Research and Development, NIFE, Stadtfelddamm 34, 30625 Hannover, Germany; 2 Unit for Reproductive Medicine — Clinic for Horses, University of Veterinary Medicine Hannover, Hannover, Germany; 3 Institute of Continuum Mechanics, Leibniz University Hannover, Hannover, Germany

**Keywords:** Biomedical engineering, Membrane biophysics

## Abstract

Cryopreservation can be used to store equine oocytes for extended periods so that they can be used in artificial reproduction technologies at a desired time point. It requires use of cryoprotective agents (CPAs) to protect the oocytes against freezing injury. The intracellular introduction of CPAs, however, may cause irreversible osmotic damage. The response of cells exposed to CPA solutions is governed by the permeability of the cellular membrane towards water and the CPAs. In this study, a mathematical mass transport model describing the permeation of water and CPAs across an oocyte membrane was used to simulate oocyte volume responses and concomitant intracellular CPA concentrations during the exposure of oocytes to CPA solutions. The results of the analytical simulations were subsequently used to develop a phenomenological finite element method (FEM) continuum model to capture the response of oocytes exposed to CPA solutions with spatial information. FEM simulations were used to depict spatial differences in CPA concentration during CPA permeation, namely at locations near the membrane surface and towards the middle of the cell, and to capture corresponding changes in deformation and hydrostatic pressure. FEM simulations of the multiple processes occurring during CPA loading of oocytes are a valuable tool to increase our understanding of the mechanisms underlying cryopreservation outcome.

## Introduction

Oocytes can be used in artificial reproduction technologies. Their longevity in vitro, however, is only limited. Cryopreservation can be used to store oocytes for extended periods at ultra-low temperatures in liquid nitrogen tanks or mechanical freezers until they are needed. Both the water-to-ice phase transition as well as the drastic temperature changes during cooling and re-warming associated with cryopreservation of cells can be very damaging^1^. Membrane permeating cryoprotective agents (CPAs) like dimethyl sulfoxide (DMSO), glycerol (GLY), or ethylene glycol (EG) need to be added to mitigate the damaging effects of ice formation and the large temperature excursions^2^. Alternatively, vitrification (ice-free cryopreservation) can be used for cryostorage, which requires a combination of high cooling rates and high CPA concentrations to completely avoid ice formation^3^. For both cryopreservation approaches, the introduction of CPAs into cells needs to be done with care, because exposing cells to CPA solutions may be toxic and cause osmotic damage.

The first step in (ice-free) cryopreservation procedures for cells is to load the cells with CPAs. Cellular membranes present a barrier for CPAs to provide intracellular protection^4^. Each cell type has its own characteristic membrane permeability characteristics that define the rate at which water and solutes (i.e., CPAs) move across the membrane. The direction of water and solute transport is governed by the direction of the osmotic and solute gradient between the intra and extra-cellular environment. Transferring cells into a solution containing molar concentrations of CPAs causes cells to respond by moving water out of the cell and CPAs into the cell. Water can pass the cellular membrane much faster compared to CPAs causing water to initially move out of the cells and a concomitant reduction in cell volume. Thereafter, both CPAs and water move into the cell until equilibrium is reached between the extra- and intracellular osmolality. Cell volume reaches a new equilibrium volume that is somewhat larger as the original volume to accommodate the CPAs initially not present inside the cell. This biphasic cell volume response during the exposure of cells to CPA solutions can be captured by video-microscopy^5–8^. Similarly, cryopreserved cells that are initially loaded with molar concentrations of CPAs after rewarming undergo swelling during CPA removal when cells are exposed to physiological media.

Different mass transport formalisms are available to fit cell volume response data to derive the characteristic cell membrane permeability to water (*L*_*p*_) and the solute (*P*_*s*_). Briefly, these include the so-called one-parameter (only water transport) or solute permeability model, the two-parameter model in which water and solute transport are considered as independent processes, and a three-parameter model which takes into account solute–solvent interactions during transport across the cellular membrane^9,10^. Once cell-specific *L*_*p*_ and *P*_*s*_ values are known, they can be used to predict volume responses during CPA loading and unloading at different CPA concentrations and, if the activation energies of water and CPA permeability are known, at different temperatures^11^.

The analytical mass transport models mentioned above do not take CPA diffusion inside the cell into account and do not provide spatial information of the complex processes associated with CPA (un)loading of oocytes. A few recently developed mathematical models, however, have implemented spatial information of intracellular CPA concentration^12,13^. The finite element method (FEM) can be used to develop phenomenological simulations of mass transport and mechanical behavior in a 3D framework^14,15^. The FEM has been used to model the response of oocytes exposed to mechanical stress in a micromanipulator set up^16^, and to simulate mechanical properties of human oocytes^17^. The FEM subdivides a system into smaller parts (the finite elements) using spatial discretization leading to the emergence of a so-called mesh that represents the finite element problem. The numerical treatment is developed based on a single element and then, it is extended to the entire discretized domain through the standard procedure of assembly based on the element connectivity.

The main aim of this study was to develop a phenomenological FEM model that can be used to design CPA loading protocols for equine oocytes. Parameters of the oocyte membrane permeability to water and EG were inferred from previously obtained video microscopic imaging results^18^. First, the two-parameter formalism was used to model the volume response and concomitant intracellular CPA concentration during the exposure of oocytes to EG solutions of varying concentrations and at different temperatures. The FEM was used to model deformation, mechanical stress, and intracellular CPA concentrations at different locations in the cell.

## Results

### Volume response and CPA uptake of oocytes during exposure to CPA solutions

**Figure 1:**
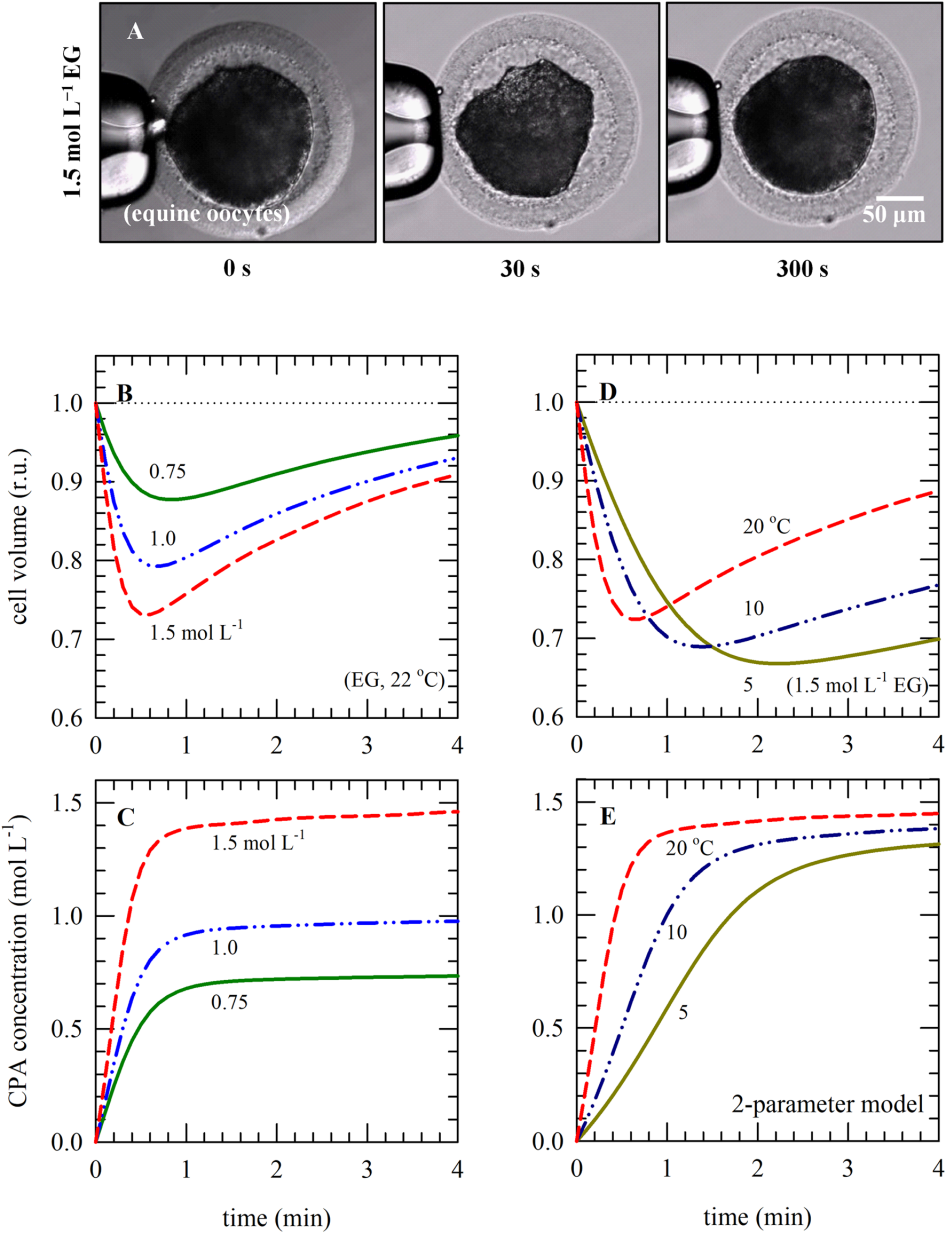
Oocyte volume responses and concomitant changes in the intracellular solute/CPA concentration during exposure to solutions containing different EG concentrations as well as temperatures. Simulations were done using the two-parameter transport model and MATLAB. Panel (**A**) illustrates the experimental setup used for investigating equine oocyte volume during exposure to various solutions, for deriving membrane permeability parameters used for the simulations depicted in panels (**B**‒**E**). Oocyte volume responses (**B**,**D**) and changes in the intracellular solute concentrations (**C**,**E**) were modeled during exposure to different EG concentrations at room temperature (**B**,**C**; 0.75 mol L^−1^: green lines, 1.0 mol L^−1^: blue lines, 1.5 mol L^−1^: red lines) as well as temperatures in case of 1.5 mol L^−1^ EG (**D**,**E**; 20 °C: red lines, 10 °C: blue lines, 5 °C: green lines).

Figure 1A shows the volume response of equine oocytes exposed to 1.5 mol L^−1^ EG at 22 °C. Cell volume response data were fitted using Eqs. (1) and (2) and the parameters listed in Table 1 to derive *L*_*p*_ and *P*_*s*_ values as previously described^18^. The membrane permeability values for water and EG and their concomitant activation energies (see Table 1) were used to simulate oocyte responses during exposure to varying EG concentrations as well as varying temperatures (Fig. 1B−E).

**Table 1:** Parameters and values used for 2-parameter fitting and modeling of equine oocyte volume responses towards solutions containing various EG concentrations.

Parameter	Description	Value	Units	Notes
*V*	Volume	(variable)	µm^3^	
*t*	Incubation duration	(variable)	µm^3^	
*M*	Molarity	(variable)	mol µm^−3^	
*T*	Temperature	278.15	K	(5 °C)
		283.15		(10 °C)
		293.15		(20 °C)
*R*	Universal gas constant	8.205745 × 10^13^	µm^3^ atm mol^−1^ K^−1^	
		1.987204	cal mol^−1^ K^−1^	
*A*	Cell area	34,763.12	µm^2^	
*V*_*0*_	Isotonic cell volume	609,469.49	µm^3^	
*V*_*bf*_	Fractional value of cell solids	0.3123	–	
*V*_*b*_	Osmotically inactive volume	190,337.32	µm^3^	
$\overline{Vs }$	Partial molar volume of permeating solute/EG	5.5919 × 10^13^	µm^3^ mol^−1^	
${M}_{n0}^{i}$	Initial cell osmolality	0.300 × 10^−15^	mol µm^−3^	
${M}_{s0}^{i}$	Initial intracellular permeating solute osmolality	0	mol µm^−3^	
${M}^{e}$	External/medium osmolality	1.800 × 10^−15^	mol µm^−3^	(1.5 mol L^−1^)
		1.000 × 10^−15^		(1.0 mol L^−1^)
		0.750 × 10^−15^		(0.75 mol L^−1^)
${M}_{n}^{e}$	External non-permeating solute osmolality	0.300 × 10^−15^	mol µm^−3^	
${M}_{s}^{e}$	Extracellular permeating solute osmolality	1.500 × 10^−15^	mol µm^−3^	(1.5 mol L^−1^)
		0.700 × 10^−15^		(1.0 mol L^−1^)
		0.450 × 10^−15^		(0.75 mol L^−1^)
*L*_*p*_	Cell membrane hydraulic permeability	0.6284	µm min^−1^ atm^−1^	* (1.5 mol L^−1^, 22 °C)
*P*_*s*_	Cell membrane solute/EG permeability	20.3660	µm min^−1^	* (1.5 mol L^−1^, 22 °C)
*E*_*Lp*_	Activation energy for *L*_*p*_	11.19 × 10^3^	cal mol^−1^	**
*E*_*Ps*_	Activation energy for *P*_*s*_	15.81 × 10^3^	cal mol^−1^	**
*L*_*pg*_	Cell membrane hydraulic permeability at 0 °C	0.1522	µm min^−1^ atm^−1^	***
*P*_*sg*_	Cell membrane solute/EG permeability at 0 °C	3.7660	µm min^−1^	***

w: water, s: permeating solute, n: non-permeating solute, o: initial value. e: extracellular, i: intracellular. *L_*p*_ and *P*_*s*_ values were experimentally derived for exposure to 1.5 M EG, at room temperature, as described previously^18^. These values were also applied for calculating volume changes in response to exposure to other EG concentrations. **The values were obtained from the literature^8^. ***Furthermore, these values were used in Eqs. (7) and (8) for deriving *L*_*pg*_, *E*_*Lp*_, as well as *P*_*sg*_ and *E*_*Ps*_ values; for calculating *L*_*p*_ and *P*_*s*_ values at different temperatures.

Oocytes exhibit a biphasic cell volume behavior when exposed to solutions containing molar concentrations of EG. First, the volume decreases, due to water moving out of the cell, where after oocytes return to their original volume due to water and solutes/EG moving into the cell. Both the initial rate of cellular dehydration as well as the minimum volume that is attained during EG exposure appears to increase with increasing EG concentrations (Fig. 1B). The minimum volume that is attained after approximately 30 s of EG exposure should not exceed the osmotic tolerance limits of the cell. The intracellular EG concentration initially shows a rapid increase coinciding with the volume decrease during EG exposure where after the intracellular EG concentration gradually approaches the extracellular EG concentration (Fig. 1C).

Temperature has a profound effect on the cell volume response and EG uptake. Both the initial rate of cell dehydration during EG exposure and the rate at which the cell returns to its original volume decrease with decreasing temperature (Fig. 1D). Moreover, the maximum extent of cell dehydration during EG exposure (i.e., the minimum volume that is attained during EG exposure) is greater at lower temperatures. The slower volume response at lower temperature coincides with a slower EG uptake and increases the time it takes to reach osmotic equilibrium (Fig. 1E).

### Use of FEM simulations to model the response of oocytes during exposure to CPA solutions

**Figure 2:**
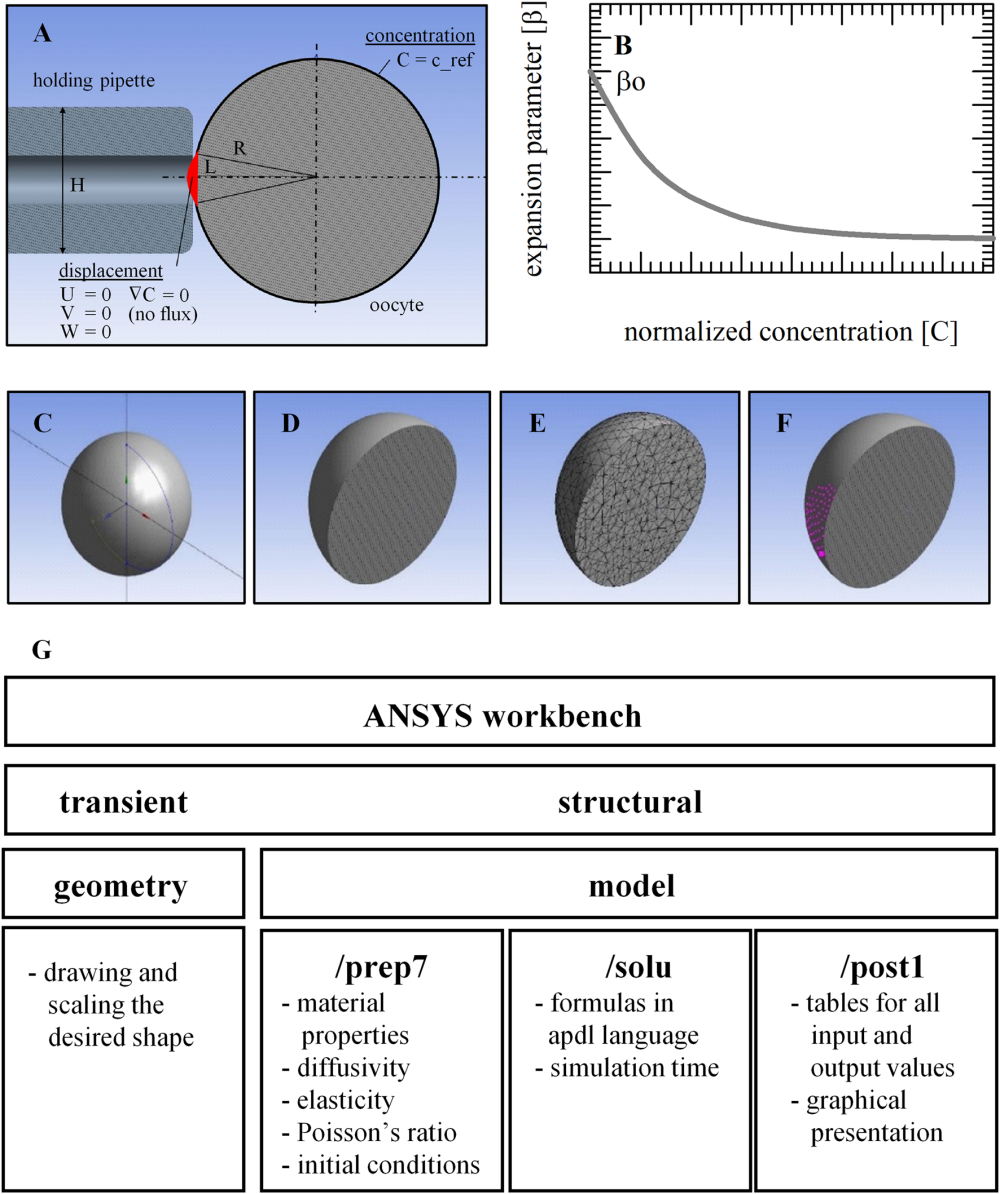
Panel (**A**) illustrates an oocyte held with a micro-capillary, as depicted in ANSYS. Volume responses that were acquired experimentally were scaled for obtaining a reference curve and deriving the expansion parameter versus the normalized concentration (**B**). In panels (**C**‒**F**) are illustrated the different views as can be obtained with finite element analysis in ANSYS, namely: cross sections at different locations (**C**,**D**), meshed shapes (**E**), and the holding point area (**F**). Panel (**G**) illustrates the ANSYS flow chart.

**Figure 3:**
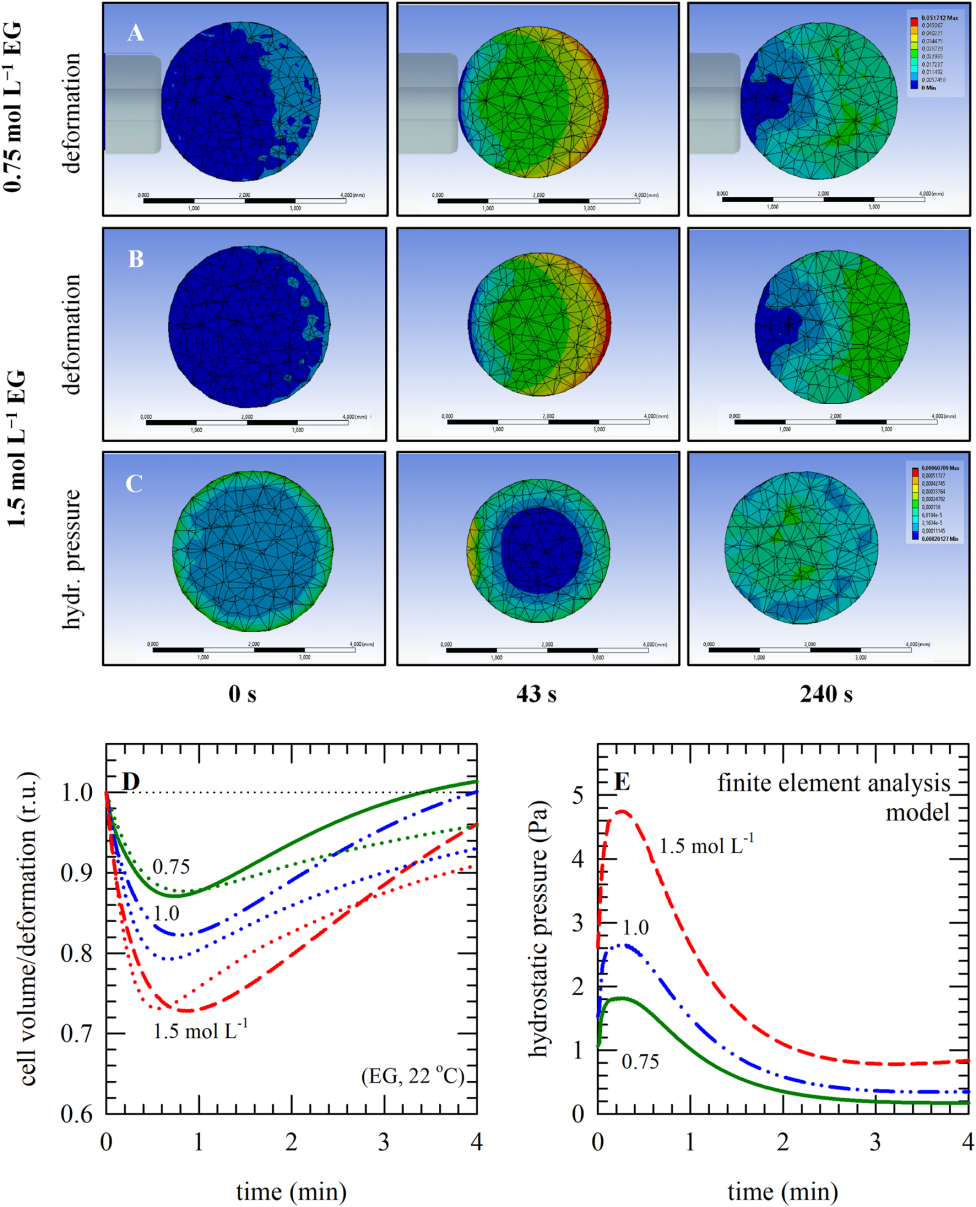
Oocyte deformation (i.e., volume changes) and associated changes in hydrostatic pressure, upon exposure to different EG concentrations, as modeled using FEM analysis in ANSYS. Panels (**A**‒**C**) depict three dimensional presentations obtained with ANSYS, illustrating oocyte deformation/volume changes (**A**,**B**), and hydrostatic pressure (**C**), in the case of exposure to 0.75 mol L^−1^ (**A**) and 1.5 mol L^−1^ (**B**,**C**) EG. In panel (**D**,**E**), respectively, average cell volume/deformation values and hydrostatic pressure values are plotted versus the exposure duration; in case of exposure to varying EG concentration at room temperature (0.75 mol L^−1^: green lines, 1.0 mol L^−1^: blue lines, 1.5 mol L^−1^: red lines). The dotted lines in panel (**D**) represent the lines obtained with the 2-parameter model as presented in Fig. 1.

Oocyte deformation (i.e., volume changes) and associated changes in hydrostatic pressure during EG exposure were modeled using FEM simulations. Figure 2A–G illustrates different views of an oocyte held with a micro-capillary as obtained with FEM analysis, and the flow chart that was used for the simulations (details are described in the ‘Materials and methods’ section). FEM simulations were done using the parameters listed in Table 2, which were optimized using the two-parameter formalism. Simulations were done for different extracellular EG concentrations to visualize cellular deformation and hydrostatic pressure in the mesh elements reflecting different cellular locations (Fig. 3A−C). The FEM simulations show a biphasic cell deformation (i.e., volume) response at different EG concentrations, which closely resemble the volume responses obtained with the two-parameter model (indicated as dotted lines in Fig. 3D). The hydrostatic pressure also shows a biphasic response; the pressure first increases to reach a concentration-dependent maximum after which the pressure gradually decreases again (Fig. 3E). The hydrostatic pressure profiles precede the deformation profiles; the maximum pressure is attained after about 15 s, whereas the minimum cell volume is attained after approximately 30 s.

**Table 2:** Parameters and coefficients used in FEM simulations.

Parameter	Description	Value	Notes
*C*_*ref*_	Concentration of the solution (maximally possible)	1.500 mol L^−1^	
		1.000 mol L^−1^	
		0.750 mol L^−1^	
*c*_*0*_	Decay constant	0.8	Estimated
*β*_*0*_	Initial expansion or contraction coefficient	− 0.20	Estimated
*D*	Diffusivity	0.0017 mm^−2^ s^−1^	From the literature^27^
*E*	Young’s modulus (modulus of elasticity)	14.52 kPa	From the lterature^26^
*ν*	Poisson’s ratio	0.49	From the literature^26^

**Figure 4:**
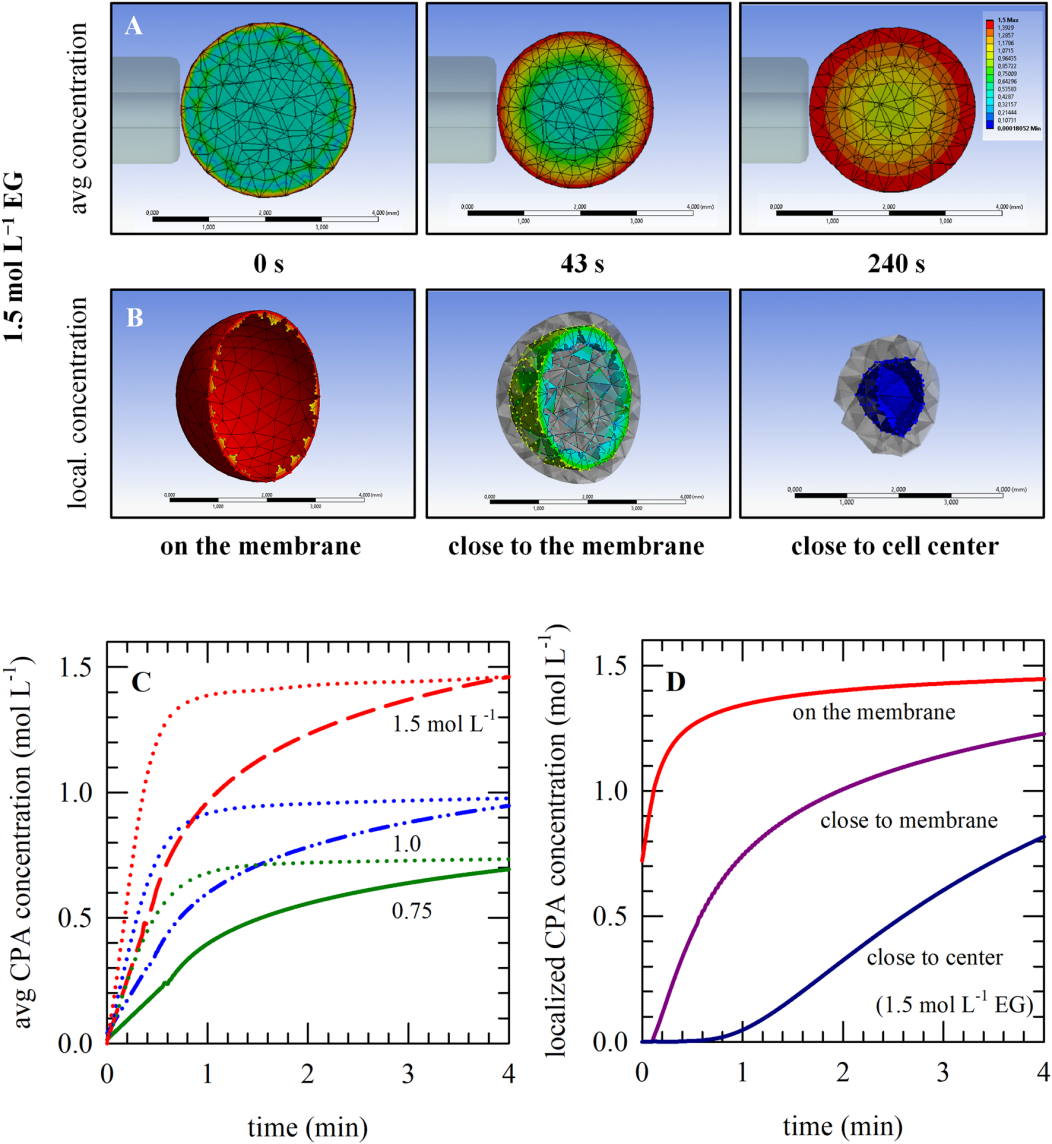
Changes in the average oocyte intracellular solute/CPA concentration as well as those at particular cellular locations. Simulations were done using a FEM model in ANSYS. Panel (**A**) represents the average intracellular CPA concentration at distinct time points during exposure to 1.5 M EG, while in panel (**B**) concentrations at particular cellular locations are shown at one particular time point (240 s). Panel (**C**) shows plots of the average CPA concentration versus time for exposure to different EG concentrations (0.75 mol L^−1^: green lines, 1.0 mol L^−1^: blue lines, 1.5 mol L^−1^: red lines). Panel (**D**) illustrates, for exposure to 1.5 mol L^−1^ EG, the change in the intracellular CPA concentration versus time at different cellular locations (on the membrane: red line, close to the membrane: purple line, close to the cell center: blue line).

Figure 4A shows FEM simulations of the spatial EG distribution in an oocyte during EG exposure. At the end of the simulation period at 4 min, the intracellular EG concentration near the membrane surface is clearly greater compared to the layers towards the center of the oocyte (Fig. 4B). Figure 4C shows that the average intracellular solute concentration during EG exposure (obtained by integrating the EG concentrations in the mesh elements depicting the oocyte) increases substantially slower versus time compared to the profile obtained using the two-parameter model (indicated as dotted lines). The two-parameter formalism only takes diffusion over the plasma membrane into account and not the EG diffusion in the cell. This explains why the kinetics of EG uptake near the membrane surface resembles the kinetics obtained by the two-parameter formalism, whereas the EG concentration versus time profiles in the layers towards the center of the cell show a much slower and more gradual increase in EG concentration over time (Fig. 4D).

**Figure 5:**
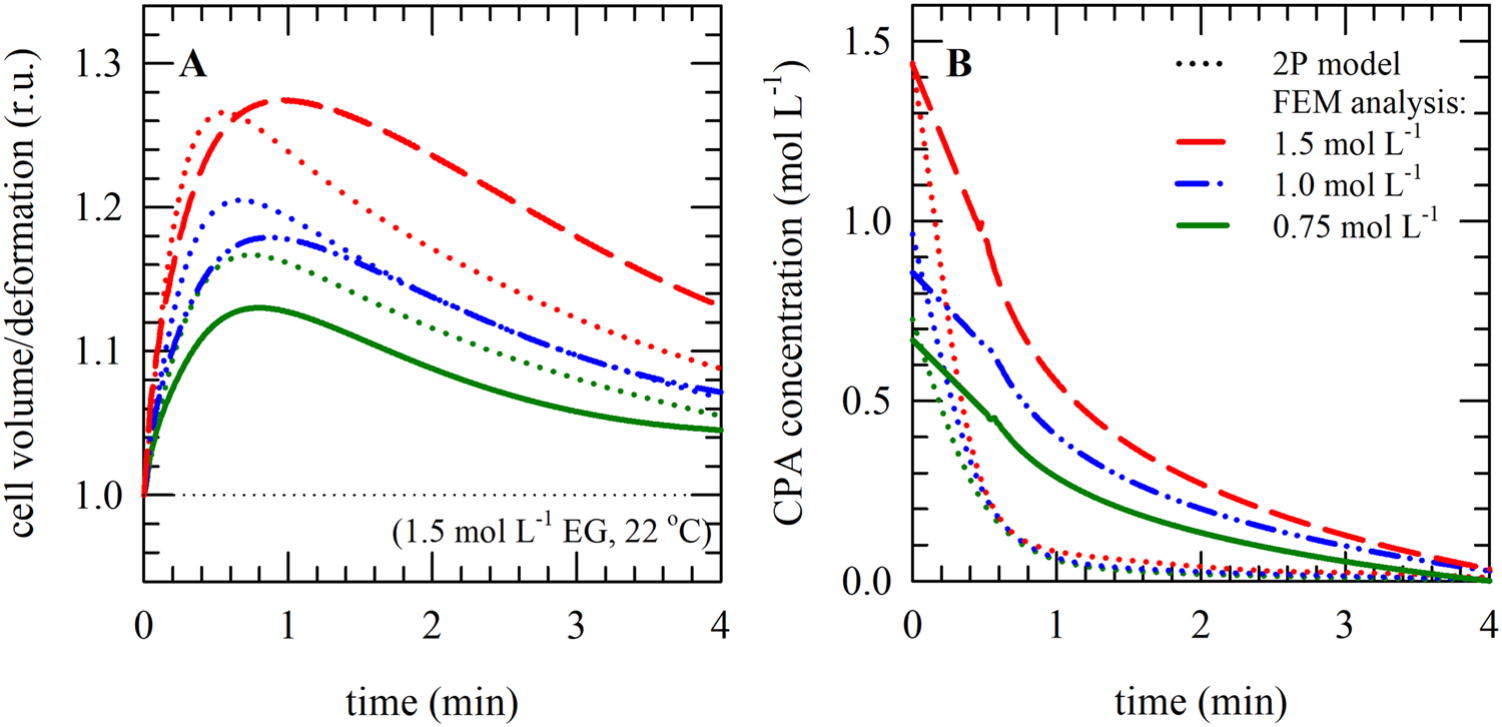
Oocyte deformation (i.e., volume changes) and associated changes in the average intracellular CPA concentration during CPA-unloading, i.e. exposure of CPA loaded cells to an isotonic solution without CPAs. Simulations were done for oocytes equilibrated in different EG concentrations (0.75 mol L^−1^: green lines, 1.0 mol L^−1^: blue lines, 1.5 mol L^−1^: red lines); cell changes/deformation (**A**) as well as the intracellular CPA concentration (**B**) have been determined versus the exposure time exposure to isotonic medium. The solid/dashed lines represent responses as obtained using FEM modeling, while the dotted lines represent responses as obtained using the 2-parameter model.

After thawing, cells are exposed to physiological solution (in vitrification procedures this is done in a step-wise fashion). The CPA that is still inside the cells result in a concentration gradient and an osmotic imbalance resulting in CPA unloading. Just like the CPA uptake, CPA unloading is also characterized by a biphasic cell volume behavior. The presence of intracellular CPAs after thawing causes an influx of water and diffusion of CPAs to the extracellular buffer. First, the volume increases, due to water moving into the cell, where after oocytes return to their original volume due to water and solutes/EG moving out of the cell (Fig. 5A). The intracellular EG concentration initially shows a rapid decrease coinciding with the volume increase during isotonic solution exposure where after the intracellular EG concentration gradually approaches zero (Fig. 5B). Just like as has been observed with CPA uptake (Fig. 4C), the CPA efflux curves obtained with the FEM simulations are delayed compared to those obtained with the two-parameter model.

**Figure 6:**
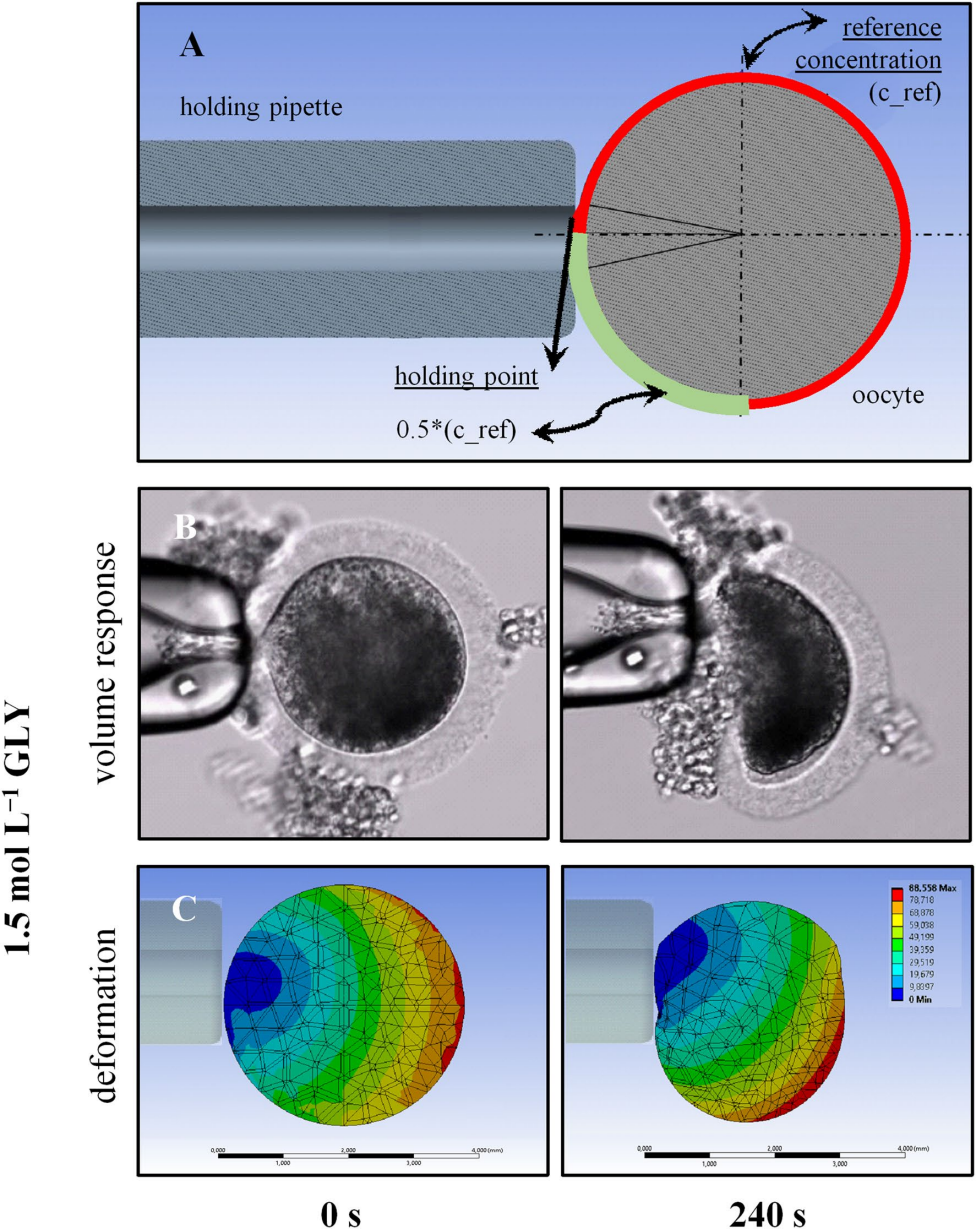
Simulation using non-symmetric boundary conditions of the CPA concentration on the exterior surface of the cell (**A**). The cell membrane is weaker or stronger in some areas leading to a non-uniform flux of both CPA and water into and out of the cell. Panel (**B**) shows the oocyte volume response upon exposure to GLY (1.5 mol L^−1^), and (**C**) shows an example of a FEM simulation using non-symmetric boundary conditions as qualitative comparison with the microscopic observations.

It is known that symmetric systems can be treated using 2D or even 1D approximations depending on the extent of symmetricity. However, this simplification is not applicable to non-symmetric cases and one needs to do a full 3D analysis in order to obtain the distribution of the variables in the entire domain. For instance, if a cell is subjected to non-symmetric boundary conditions, one expects to get a non-symmetric response. This can be simulated using non-symmetric boundary conditions of the CPA concentration on the exterior surface of the cell (Fig. 6A). The boundary conditions for this test case are essentially similar to those of the symmetric case shown in Fig. 2A except for the solute concentration at the exterior surface. One can see that part of the cell (shown in light green in Fig. 6A) is exposed to a lower concentration (0.5 *C*_*ref*_) while the rest of the cell surface is exposed to the maximum reference concentration *C*_*ref*_ (shown in dark green in Fig. 6A). From a physical point of view, this example mimics a situation in which the permeability of the cell membrane is weaker or stronger in some areas leading to a non-uniform flux of both CPA and water into and out of the cell. Particularly with glycerol, which has a relatively low *P*_*s*_, non-uniform shrinkage has been observed (Fig. 6B). The FEM simulation (Fig. 6C) shows a qualitative comparison with the microscopic observations.

## Discussion

Analytical mass transport models have been widely used to simulate cell volume responses and CPA uptake during the exposure of cells to CPA solutions^5,6,8,19^. Modeling predictions can be used to avoid conditions that cause cells to shrink or swell beyond their osmotic tolerance limits and to compute the time needed to reach a sufficiently high enough intracellular CPA concentration to protect against freezing injury^11^. For vitrification approaches modeling can be used to estimate the time needed to reach maximal dehydration of the cell prior to cooling, while keeping the exposure time as short as possible to reduce toxicity effects^20^. Precise timing is of the essence to maximize the cryopreservation outcome.

Exposing cells to anisotonic solutions above or below their tolerance limits results in a rapid decline in cell viability. The osmotic tolerance limits define the maximum tolerable volume excursions that a cell can withstand via the Boyle van’t Hoff equation. For red blood cells, cell volume excursions should be maintained below 1.7 times the isotonic osmotically active volume to avoid hemolysis^21^.

Oocyte deformation is a major cause of potential cell damage which can lead to disruption of the spindle apparatus and failure in embryo development after in vitro fertilization^22,23^. Therefore, for oocytes, the osmotic tolerance limits are not only defined by the apparent membrane integrity after exposure to anisotonic solutions, but also by their developmental potential. Porcine and equine oocytes respond as linear osmometers when they are exposed to anisotonic solutions ranging from, respectively, 180 to 800 mOsm kg^−1^ and 200 to 600 mOsm kg^−1^ suggesting that they can tolerate exposure to anisotonic solutions in these osmotic ranges, i.e., the oocyte membrane remains intact^4,18^. However, damage in the oocyte spindle apparatus only becomes apparent in its developmental potential, i.e., the ability of an oocyte to form blastocysts after in vitro fertilization or intracytoplasmic sperm injection. A study on porcine oocytes showed that only about half of the oocytes maintain their developmental potential after exposure to anisotonic solutions only slightly above or below isotonic conditions^24^.

In the 2-parameter formalism, only the plasma membrane (i.e., outer membrane) is included as diffusion barrier. As a consequence this model yields a rapid increase of the intracellular CPA concentration reaching a plateau within 30−60 s. The intracellular environment is not solely comprised of fluid, but also includes organelles all surrounded by their own membranes (i.e., apolar diffusion barriers). Use of the FEM model actually may give a more realistic estimation of the intracellular CPA concentration during CPA loading, namely a slower increase in intracellular CPA concentration. For cryopreservation purposes, the mechanical response of the cell is particularly important, i.e., the time it takes for the cell volume to attain equilibrium again. The simulations show that the mechanical response of oocytes during CPA exposure (shrinkage followed by swelling) actually is not yet fully completed in 4 min.

The two-parameter formalism only requires knowledge of the membrane permeability characteristics and dimensions of the cell. However, exposing a cell to osmotic imbalance causes a complex series of events including deformation as well as mass transport in the extracellular space, across the cell membrane and within the intracellular spaces. The FEM was used here to develop a phenomenological continuum model to simulate strain–stress behavior of an oocyte kept in position by a holding pipette during exposure to a CPA solution without using analytical solutions and prior knowledge of molecular and microscopic structures. The parameters used in the FEM model were optimized using the two-parameter formalism so that the characteristic biphasic cell volume behavior of both models resembled each other, even though the volume responses were not identical. Simulations of the EG concentrations in the mesh elements depicting the oocyte provided insights in the kinetics of the CPA loading process in different cellular locations of the cell. Close to the membrane surface, the uptake kinetics resembled the kinetics obtained using the two-parameter formalism, whereas the uptake in locations closer to the center of the cell, show a delayed and slower CPA uptake kinetics different as predicted by the two-parameter formalism. The FEM model was developed for EG, but can easily be modified for other CPAs by changing the parameters in the FEM algorithm, particularly the diffusivity and the initial expansion or contraction coefficient.

Summarizing, whereas analytical transport models can be used to simulate average oocyte volume responses and concomitant intracellular CPA concentrations during exposure of oocytes to CPA solutions, FEM models provide more detailed and complementary information of the CPA loading process. FEM simulations can be used to depict spatial differences in CPA concentration during CPA permeation and to capture corresponding changes in deformation and hydrostatic pressure. Modeling the response of oocytes exposed to CPA solutions can be used to define suitable conditions to safely load oocytes with CPAs, and to remove the CPAs again after thawing.

## Conclusion

The objective of this 3D continuum model is to perform a full analysis of the oocyte response in the presence of a CPA solution. One can extend the model and incorporate thermal effects on intracellular CPA distribution during freezing or thawing. Spatial information on local CPA concentrations can in turn be used to predict the likelihood of intracellular vitrification and/or intracellular ice-formation. Such models are particularly useful if they are supported and verified by microscopy results. Once the model is validated, it can be used to conduct in silico experiments that can be used to optimize cryopreservation or vitrification protocols for cells, which is substantially cheaper than in vitro investigations. In our upcoming work, we plan to incorporate local structures including the plasma membrane and organelles in conjunction with the diffusion process within the bulk of the cell. The explicit modelling of water content using ‘porous media framework’ as well as ‘thermal effects’ can also be introduced into the model.

## Materials and methods

### Experimental assessment of oocyte membrane permeability parameters

Data from a previous study^18^ were used to develop analytical and FEM models that can be used to simulate oocyte volume responses during exposure to CPA solutions. In this previous study^18^, equine oocyte volume responses were analyzed upon perfusion with saline supplemented with 1.5 mol L^−1^ permeating cryoprotective agent (i.e., ethylene glycol/EG or glycerol/GLY). Volume responses were recorded and analyzed, using an inverted microscope with a micromanipulator setup. Briefly, oocytes were transferred to a 10 μL droplet of isotonic medium on a culture dish, which was placed on the microscope-micromanipulator stage. A holding capillary was used with enough negative pressure to hold the oocyte at a fixed position. A syringe was used for adding 1.5 mL CPA solution onto the droplet in which the oocyte was held.

The two-parameter transport formalism^10^ was used to fit oocyte volume versus time plots to derive the membrane permeability to water (*L*_*p*_) and for EG (*P*_*s*_) as described in our previous paper^18^. Fitting was done using MATLAB software (Mathworks, Natick, MA, USA). The cell volume (*Vc*) change during the perfusion process, at a particular temperature (*T*), can be described as a function of the water and solute volume (*V*_*w*_ and *V*_*s*_, respectively):

1 $$\frac{dVw}{dt}= -{L}_{p}\times A\times R\times T\times \left({M}^{e}-{M}^{i}\right),$$

2 $$\frac{dVs}{dt}={P}_{s}\times A\times \left({M}_{s}^{e}-{M}_{s}^{i}\right)\times \overline{{V }_{s}},$$

furthermore:

3 $${V}_{c}= \left({V}_{w}+{V}_{s}+{V}_{b}\right),$$

4 $${M}^{i}= \left({M}_{n}^{i}+{M}_{s}^{i}\right),$$

5 $${M}_{n}^{i}=\frac{{M}_{n0}^{i}\times \left(\left(1-{V}_{bf}\right)\times {V}_{0}\right)}{{V}_{w}},$$

6 $${M}_{s}^{i}=\frac{{V}_{s}}{\overline{{V }_{s}}\times {V}_{w}},$$

where, $\overline{{V }_{s}}$ is the partial molar volume of the CPA, M the osmolality, w refers to water, s to permeating and n to non-permeating solute, 0 refers to the initial value, while e and i refer to external and internal cellular locations, respectively. *R* represents the universal gas constant and cell specific parameters include the area (*A*), isotonic volume (*V*_*0*_), the osmotically inactive volume (*V*_*b*_), and osmotically active volume ((1 − *V*_*bf*_) × *V*_*0*_) with *V*_*bf*_ referring to the fractional value of cell solids. It was assumed that CPA solutions behave as ideal solutions.

The temperature dependence of the transport parameters can be described using the following Arrhenius relationship:

7 $$ln\left({L}_{p}\right)=ln\left({L}_{p}\right)-\left(\frac{{E}_{Lp}}{R}\right)\times \left(\frac{1}{T}-\frac{1}{{T}_{0}}\right),$$

8 $$ln\left({P}_{sg}\right)=ln\left({P}_{sg}\right)-\left(\frac{{E}_{Ps}}{R}\right)\times \left(\frac{1}{T}-\frac{1}{{T}_{0}}\right),$$

where, rates and temperatures are given per s and in K, respectively, while *L*_*pg*_ and *P*_*sg*_ are the reference values of *L*_*pg*_ and *P*_*sg*_ at the reference temperature *T*_*0*_ (i.e., 273.15 K which equals 0 °C), and *E*_*Lp*_ and *E*_*Ps*_ represent the activation energies for water and solute (CPA) transport across cell membrane, respectively.

### Modeling oocyte volume responses and intracellular CPA concentrations during exposure to different CPA concentrations and temperatures

Membrane transport parameters, *L*_*p*_ and *P*_*s*_, of equine oocytes were previously studied using the oocyte volume response upon exposure to 1.5 mol L^−1^ EG^18^. The obtained *L*_*p*_ and *P*_*s*_ parameters were used to simulate oocyte volume responses and concomitant intracellular CPA concentrations upon exposure to varying EG concentrations and at different temperatures. A detailed listing of the parameters and values that were used for modeling equine oocyte volume responses and concomitant intracellular solute concentrations according to the two equations of the two-parameter formalism is presented in Table 1. A user-friendly interface was created with the App-designer included in MATLAB.

The code includes a section for calculating the *L*_*p*_ and *P*_*s*_ values at varying temperature (according to Eqs. 7 and 8):

9 $${L}_{p} = {L}_{pg}*exp\left(\left(-\frac{{E}_{{L}_{P}}}{R}\right)*\left(\left(\frac{1}{T}\right)-\left(\frac{1}{{T}_{0}}\right)\right)\right),$$

10 $${P}_{s}= {P}_{sg}*exp\left(\left(-\frac{{E}_{{P}_{s}}}{R}\right)*\left(\left(\frac{1}{T}\right)-\left(\frac{1}{{T}_{0}}\right)\right)\right),$$

furthermore, it is linked to an Excel data sheet from which the following parameters are imported:

${L}_{pg}$ = cell membrane hydraulic permeability (*L*_*p*_) value at 273.15 K.

${P}_{sg}$ = cell membrane solute permeability (*P*_*s*_) value at 273.15 K.

${E}_{{L}_{P}}$ = activation energy of cell membrane hydraulic permeability.

${E}_{{P}_{s}}$ = activation energy of cell membrane solute permeability.

*R* = universal gas constant.

*T* = room temperature.

*T*_*0*_ = initial temperature (0 °C = 273.15 K).

In a separate section, the oocyte volume responses versus time are then calculated using:

11 $${V}_{b} = {V}_{0}*{V}_{bf},$$

12 $${V}_{w0} = {V}_{0}-{V}_{b},$$

13 $$f = @(t,x)[-{L}_{p}*A*R*T*({M}_{e}-x(2)/)({V}_{s}^{0}*x(1))-{M}_{{n}_{0}}^{i}*\left(1-{V}_{bf}\right)*{V}_{0}/x(1)); {V}_{S}^{0}*{P}_{s}*A*({M}_{s}^{e}-x(2)/({V}_{s}^{0}*x(1)))],$$

14 $$\left[t,x\right]= ode113\left(f,{t}_{s},{x}_{0}\right),$$

where:

*V*_*w0*_ = isotonic (initial) water volume.

*V*_*b*_ = osmotically inactive cell volume.

*V*_*0*_ = isotonic (initial) cell volume.

*V*_*bf*_ = osmotically inactive cell volume fraction.

$${M}_{e}$$ = external osmolality.

${V}_{s}^{0}$= partial molar volume of solute.

${{M}_{n}^{i}}_{0}$ = initial cell osmolality.

${M}_{s}^{e}$ = external solute osmolality.

*x(1)* = volume of water (*V*_*w*_).

*x(2)* = volume of permeating solute/EG (*V*_*s*_).

The concomitant intracellular solute concentration versus time is calculated via:

15 $${M}_{s}^{i} = {V}_{s}/({V}_{s}^{0}*{V}_{w}),$$

16 $${V}_{n}=(({V}_{w}+{V}_{s}+{V}_{b})/{V}_{0}),$$

where: ${M}_{s}^{i}$ = internal permeating solute osmolality, ${V}_{n}$ = normalized volume.

Finally, the numeric data of the calculated cell volume and solute concentration versus time plots are exported to an Excel data sheet, and plots were created using Sigmaplot for Windows version 13.0 (Systat Sotware Inc., http://www.systat.de/index.html).

### Modeling of diffusion-induced deformation of oocytes during exposure to different CPA concentrations using ANSYS

Using ANSYS (ANSYS Workbench, academic license version 19.2, https://www.ansys.com/), as a solver based on FEM, simulations were conducted in which cell volume change is considered to be the result of diffusion-induced mechanical deformation. The cell volume change, from a physical point of view, originates from variations in the cell water content which is dictated by the cell membrane permeation and osmotic pressure. In our model, we only took into account the diffusion of the solute, whereas the osmotically driven water flow was not explicitly incorporated in the model. Nevertheless, the indirect and implicit impact of the volume change due to the cellular water content was introduced into the deformation process. It is a phenomenological model in which one can interpret the volume change of the cell as a process depending on the concentration of the solute, namely CPA. As a matter of fact, the dependency of water content, as the one that is directly responsible for the volume change, on the CPA concentration plays the key role. If we were to account for the water ‘explicitly’, we would have to extend the model to a poro-elastic one in the framework of porous media theory. Hence, for the sake of simplicity, we exclude this part by assuming a phenomenological model and directly connecting the CPA concentration to the local volume change (mechanical strain). The coupling between the deformation and the diffusion-driven expansion was feasible through developing a computer code using ANSYS Parametric Design Language (APDL)^25^.

The applied model is transient, meaning that variables are dependent on not only the position but also the time. The inertial effects (i.e., mechanical acceleration), however, were not taken into account due to the fact that the process is relatively slow compared to systems without an internal damping effect (e.g., a load in static structural analyses does not change or changes slightly that do not disrupt the steady-state conditions) and hence dynamics does not play a role here. Unlike mechanical processes, the solute diffusion phenomenon is time-dependent.

Appropriate boundary conditions were chosen to mimic the experimental set up. Two field variables are considered, namely mechanical deformation and CPA concentration. The oocyte is held at a specific region using a capillary with a defined diameter, while the solution containing specific solutes/CPAs is added (Fig. 2A). From a mathematical point of view, the oocyte is mechanically subjected to so-called Dirichlet boundary conditions. It means that the deformation components are set to zero at the zone kept by the capillary (indicated in red in Fig. 2A). The rest of the cell is free to undergo deformation due to swelling/shrinkage. Similar to the mechanical field, the boundary condition of the diffusion equation is of Dirichlet type at the regions submerged in the CPA solution. It means that the maximum solute concentration (denoted by *C*_*ref*_) is prescribed on the exterior surface of the cell (shown in dark green in Fig. 2A). Nevertheless, in the cellular region that is in contact with the capillary (shown in red in Fig. 2A), a zero flux condition is enforced. In mathematical terms, it corresponds to a Neumann type boundary condition in which the concentration gradient is prescribed.

The simulation procedure can be divided into three sections, namely: pre-processing, solving and post-processing modules. In the pre-processing part, the following inputs are defined: the initial conditions, types of elements used, diffusion coefficients, saturation and reference concentrations, the elasticity module and Poisson’s ratio, and diffusion expansion coefficients. The solid227 element was chosen to perform combined structural diffusion analysis. When used in coupled-field analyses having structural and diffusion DOFs; this element has ten nodes with up to six degrees of freedom per node and supports the effects of diffusion strain and hydrostatic stress migration (i.e., transport of particles due to a hydrostatic stress gradient).

In the solving-section of the program, first the boundary conditions are defined and it will be determined how long the simulation will take. For being able to compare the simulation with the experimental data (i.e., oocytes were kept in place with a holding capillary), some points of the sphere were fixed resulting in a restricted shape change. A summary of the mathematical framework behind the model is described hereafter. As a convention, unlike scalar variables, vectorial variables are represented in bold, while the matrices are distinguished using squared brackets.

In a coupled structural-diffusion analysis, the total strain ${\varvec{\varepsilon}}$, that is represented in vectorial Voigt notation, is composed of elastic ${{\varvec{\varepsilon}}}^{el}$ and ${{\varvec{\varepsilon}}}^{di}$ diffusion parts, respectively:

17 $${\varvec{c}}={{\varvec{\varepsilon}}}^{el}+{{\varvec{\varepsilon}}}^{di}={\left[{\varvec{E}}\right]}^{-1}{\varvec{\sigma}}+{\varvec{\beta}}\left(C-{C}_{ref}\right),$$

where:

***ε*** = total strain vector = [*ε*_*x*_* ε*_*y*_* ε*_*z*_* ε*_*xy*_* ε*_*yz*_* ε*_*xz*_]^*T*^*.*

${{\varvec{\varepsilon}}}^{el}$= elastic strain vector.

${{\varvec{\varepsilon}}}^{di}$ = diffusion strain vector.

${\varvec{\sigma}}$ = stress vector = [*σ*_*x*_* σ*_*y*_* σ*_*z*_* σ*_*xy*_* σ*_*yz*_* σ*_*xz*_]^*T*^*.*

*C* = concentration field.

*C*_*ref*_ = reference concentration.

$${[{\varvec{E}}]}^{-1}=\frac{1}{E}\left[{\begin{array}{c}1\\ -\nu \\ -\nu \\ 0\\ 0\\ 0\end{array} \begin{array}{c} -\nu \\ 1\\ -\nu \\ 0\\ 0\\ 0\end{array} \begin{array}{c} -\nu \\ -\nu \\ 1\\ 0\\ 0\\ 0\end{array} \begin{array}{c} 0 \\ 0\\ 0\\ 2(1+\nu )\\ 0\\ 0\end{array}\begin{array}{c}0\\ 0 \\ 0\\ 0\\ 2(1+\nu )\\ 0\end{array}\begin{array}{c}0\\ 0\\ 0\\ 0\\ 0\\ 2(1+\nu )\end{array}}\right]$$ = inverse of elastic stiffness matrix with *E* being the Young’s modulus and ν the Poisson’s ratio.

${\varvec{\beta}}$ = vector of coefficients of diffusion expansion = [*β*_*x*_* β*_*y*_* β*_*z*_* 0 0 0*]^*T*^*.*

Here, ${\left[{\varvec{E}}\right]}^{-1}{\varvec{\sigma}}$ is mechanical deformation and ${\varvec{\beta}}(C-{C}_{ref}$) is diffusion-induced swelling/shrinking. Assuming an isotropic behavior, *β*_*x*_* , β*_*y*_ and *β*_*z*_ are taken to be identical and equal to *β* meaning that *βx* = *βy* = *βz* = *β*.

The mechanical strain is defined using the gradient of displacement vector (denoted by ***u***) as follows:

18 $${\varvec{\varepsilon}}=\frac{1}{2}.\left(\nabla {\varvec{u}}+{\nabla {\varvec{u}}}^{T}\right),$$

in which the Nabla operator (∇) signifies the spatial gradient.

Assuming a simple Fickian transport, the mass flux vector ${\varvec{J}}$ for the diffusion of the solute is described by:

19 $${\varvec{J}}=-\left[{\varvec{D}}\right] \nabla C,$$

where ${\varvec{J}}$ represents the diffusion flux density and **[*D*]** as the diffusivity matrix is defined according to:

20 $$\left[{\varvec{D}}\right]=\left[\begin{array}{ccc}{D}_{XX}& 0& 0\\ 0& {D}_{YY}& 0\\ 0& 0& {D}_{zz}\end{array}\right],$$

in which ${D}_{XX}$, ${D}_{YY}$, and ${D}_{zz}$ are diffusivity coefficients in the X, Y, and Z directions, respectively. Similar to the diffusion expansion coefficient, the diffusivity coefficients are taken to be identical and consequently it leads to an isotropic diffusive behavior, namely ${D}_{XX}$ =*D*_*YY*_ = *D*_*ZZ*_ = *D*.

It is assumed that the diffusion expansion parameter *β* is a function of the solute concentration which controls the water content. It is obvious that the local volume of a material point changes due to a change in the volume fraction of the water there. Since the osmotic flow of the water is not explicitly present in our model, the diffusion expansion parameter is directly assumed to be a function of the solute concentration. For the sake of simplicity, this dependency is assumed to be of the exponential type according to:

21 $$\beta ={\beta }_{0}.{e}^{(\frac{-\overline{C}}{{C }_{0}})},$$

where ${\beta }_{0}$ and ${C}_{0}$ describe the dependence of $\beta$ versus dimensionless concentration $\overline{C }$.

It should be noted that $\overline{C }$ is a dimensionless representation of the solute concentration according to:

22 $$\overline{C }=\frac{C}{({C}_{ref}-C)},$$

Figure 2B illustrates the variation of $\beta$ as a function of $\overline{C }$.

One should notice that Eq. (21) is only a choice that contains two meaningful parameters: Initial value (${\beta }_{0}$) and decay rate (*C*_*0*_). Here, the water content variation as the main driver of swelling/shrinkage is translated into the evolution of parameter *β*. From a physical point of view, the water content depends on the availability of CPA concentration, namely *β* = *β(C)*. Since the CPA concentration at any point is a function of time due to the transient diffusion equation, namely *C* = *C(x,t)*, it implies that the parameter *β* is a function of time. The overall volume of the cell changes in the course of time. The volume simulations obtained using the 2-parameter formalism form the basis to develop the FEM model. Hence, ${\beta }_{0}$ and ${C}_{0}$ have been calibrated using the volume curves obtained with the 2-parameter formalism, which in turn are based on experimentally determined volume response curves of oocytes exposed to a 1.5 mol L^−1^ EG solution^18^ (i.e., relative cell volume change versus time plots in Fig. 3D for 1.5 mol L^−1^). In other words, the optimal value for these two parameters are found in such a way that fit experimental observations of the volume change over time. Once ${\beta }_{0}$ and ${C}_{0}$ have been estimated, one can use them similar to other invariant parameters (*E*,*ν*,*D*) in order to predict the behavior of the system for other conditions.

The balance of mass for the solute concentration *C* is described by the following diffusion equation:

23 $$\dot{C}=\nabla .\mathbf{J},$$

where the overhead dot stands for time derivative and the symbol *∇*. is defined as the divergence operator. In addition to the balance of mass, one should take into account the equilibrium of mechanical forces that is governed by:

24 $$\nabla .{\varvec{\upsigma}}=0,$$

where ${\varvec{\upsigma}}$ signifies the mechanical stress tensor defined in Eq. (17). It is apparent that, unlike Eq. (23), the mechanical governing Eq. (24) is assumed to be time-independent.

Applying the variational principle to the structural (Eq. 24) and diffusion equations (Eq. 23) coupled via the constitutive equations (Eqs. 17 and 19), one can obtain the following finite element matrix equations for the structural-diffusion analysis:

25 $$\left[\begin{array}{cc}\left[0\right]& \left[0\right]\\ \left[0\right]& \left[{C}^{d}\right]\end{array}\right]\left\{\begin{array}{c}\dot{{\varvec{u}}}\\ \dot{C}\end{array}\right\}+\left[\begin{array}{cc}\left[{K}^{u}\right]& \left[{K}^{ud}\right]\\ \left[{K}^{du}\right]& \left[{K}^{d}\right]+\left[{K}^{dN}\right]+\left[{K}^{dc}\right]+\left[{K}^{dcN}\right]\end{array}\right]\left\{\begin{array}{c}{\varvec{u}}\\ \mathrm{C}\end{array}\right\}=\left\{\begin{array}{c}\mathbf{F}\\ R\end{array}\right\} ,$$

where:

[${K}^{u}$] = element stiffness matrix.

$\dot{{\varvec{u}}}$ = nodal displacement vector.

$\mathbf{F}$ = sum of the element nodal force and element pressure vectors.

[${C}^{d}$] = element diffusion damping matrix.

[${K}^{d}$] = element diffusion conductivity matrix.

*C* = nodal concentration vector.

$\dot{C}$ = time derivative of nodal concentration vector.

*R* = nodal diffusion flow rate vector.

[${K}^{ud}$] = element diffusion strain stiffness matrix.

[${K}^{du}$] = element transport conductivity matrix.

[${K}^{dN}$] = nonlinear part of the element diffusion conductivity matrix.

[${K}^{dc}$] = element conductivity matrix associated with diffusion strain.

[${K}^{dcN}$] = nonlinear part of the element conductivity matrix.

The nodal displacement vector ***u*** and nodal concentration *C* are the unknowns in the finite element problem in hand. On the other hand, the sum of the element nodal force ***F*** and nodal diffusion flow rate vector *R*, which constitute the right hand side of the finite element formulation, are known due to the prescribed boundary conditions. Figure 2C‒F illustrate the different views that can be obtained with finite element analysis in ANSYS, namely: cross sections at different locations (Fig. 2C,D), meshed shapes (Fig. 2E), and the holding point area (Fig. 2F). In the post-processing section of the program, one can generate graphical outputs of the simulation such as contours and animations. The ANSYS workflow is illustrated in Fig. 2G. The ANSYS model is based on 5 parameters: *E*, *ν*, *D*, *β*_*0*_, *C*_*0*_ reported in Table 2. Because experimentally captured oocyte volume excursions took place during a 4 min time interval^18^, we kept this time for the ANSYS simulations for concurrent evaluation of cell deformation and CPA concentration.
